# Wild bats briefly decouple sound production from wingbeats to increase sensory flow during prey captures

**DOI:** 10.1016/j.isci.2021.102896

**Published:** 2021-07-22

**Authors:** Laura Stidsholt, Mark Johnson, Holger R. Goerlitz, Peter T. Madsen

**Affiliations:** 1 Zoophysiology, Department of Biology, Aarhus University, Aarhus, Denmark; 2 Aarhus Institute of Advanced Studies, Aarhus University, Aarhus, Denmark; 3 Acoustic and Functional Ecology, Max Planck Institute for Ornithology, Seewiesen, Germany

**Keywords:** biological sciences, ecology, environmental science, ethology, zoology

## Abstract

Active sensing animals such as echolocating bats produce the energy with which they probe their environment. The intense echolocation calls of bats are energetically expensive, but their cost can be reduced by synchronizing the exhalations needed to vocalize to wingbeats. Here, we use sound-and-movement recording tags to investigate how wild bats balance efficient sound production with information needs during foraging and navigation. We show that wild bats prioritize energy efficiency over sensory flow when periodic snapshots of the acoustic scene are sufficient during travel and search. Rapid calls during tracking and interception of close prey are decoupled from the wingbeat but are weaker and comprise <2% of all calls during a night of hunting. The limited use of fast sonar sampling provides bats with high information update rates during critical hunting moments but adds little to their overall costs of sound production despite the inefficiency of decoupling calls from wingbeats.

## Highlights

• Wild bats decouple wingbeats and call emission in short critical phases of hunting
• These rapid capture calls are more than 30 dB weaker than most other calls
• Fast sonar calls are vital for hunting but comprise just 2% of all emitted calls
• High sensory flow when hunting therefore adds little to the cost of sound production

## Graphical abstract

**Figure:**
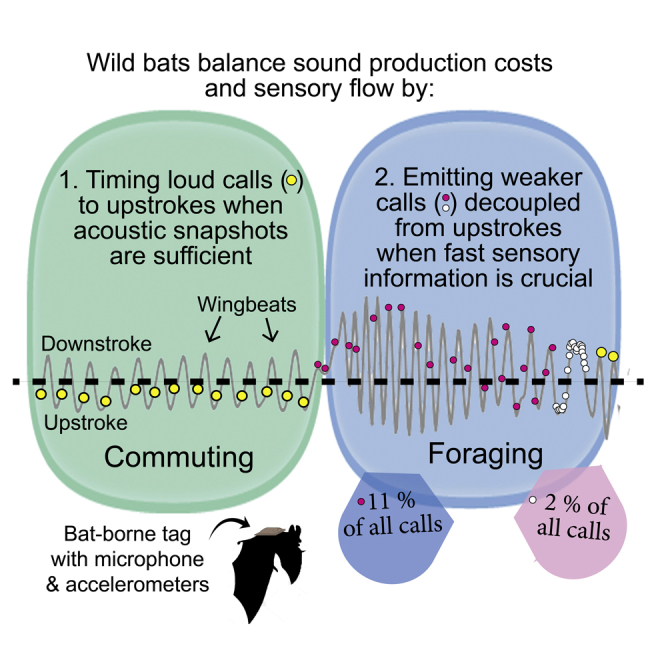

## Introduction

Animals balance a trade-off between acquiring sufficient sensory information from their surroundings and the costs of doing so (Laughlin, 2001). For most animals, this cost is primarily due to the neural processing of passive sensory signals and high maintenance costs of sensory and nervous systems (Laughlin, 2001). However, for active sensing animals, the cost of sensory acquisition is also influenced by the production and implementation of the energy needed to investigate their environment (Nelson and MacIver, 2006). For example, electric fish have converted a large part of their swimming muscles to electrocytes and tilt their body when capturing prey to improve their active sensing performance at the cost of a reduction in swimming efficiency (MacIver et al., 2010). Toothed whales use pneumatic sound production in their nasal complex to generate powerful clicks with very small air volumes that must be recycled after a series of clicks. Toothed whales must therefore choose between emitting numerous and weak or fewer and loud clicks per recycling (Foskolos et al., 2019), and all but the sperm whale cannot breathe and click at the same time (Wahlberg et al., 2005). To the contrary, echolocating bats must breathe to vocalize (Speakman and Racey, 1991). Like most other terrestrial mammals that synchronize breaths with locomotory strides (Bramble, 1989), bats generally produce echolocation calls on exhalations that are synchronized with each upstroke of their wingbeat cycle (Koblitz et al., 2010; Suthers et al., 1972) to make an otherwise expensive sound production much cheaper (Lancaster et al., 1995; Speakman and Racey, 1991). The maximum vocal output is achieved at the top of the upstroke when abdominal muscle force to produce the downstroke is likely maximal (Koblitz et al., 2010). Despite this coupling, a recent laboratory study measured strongly increasing metabolic costs for the production of calls with source levels above 110 dB root mean square (RMS) at 0.1 m (Currie et al., 2020). Sound production at other times in the wingbeat cycle leads to lower call levels and presumably to less efficient sound production (Koblitz et al., 2010). However, depending on the behavioral task, bats can break the tight relationship between sound emission and wingbeats by emitting calls throughout the entire wingbeat cycle as observed in the laboratory (Lancaster et al., 1995; Moss et al., 2006) and field (Kalko and Schnitzler, 1989). In one captive study, *Eptesicus fuscus* emitted calls throughout the entire wingbeat cycle, leading to the suggestion that the bats' vocal control could override the wingbeat cycle during the buzz, but no direct quantification of the relationship was established (Moss et al., 2006). Thus, although the decoupling between wingbeats and buzz calls is widely assumed to take place (Kalko, 1994; Kalko and Schnitzler, 1989; Koblitz et al., 2010; Lancaster et al., 1995; Suthers et al., 1972), the precise relationship between buzz call timings, levels, and the wingbeat phase remains to be understood and quantified. Moreover, bats may echolocate differently in the wild as compared to controlled laboratory settings. For example, bats can call up to 20 dB louder in the wild and yet must navigate and catch food in complex environments likely calling for a more adaptive relationship between wingbeat, respiration, and biosonar sampling. So far, little is known about when and how often echolocating bats dispense with cheap sound production to improve sensory flow over a range of echolocation behaviors in the wild.

In the wild, many species of echolocating bats fly long distances every night to their foraging habitats (spanning 9.8 to 53.5 km for five different species (Egert-Berg et al., 2018)), where they spend additional time on the wing to catch prey. To meet the energy requirements of such long flights, bats must strive for a low cost of transport and at the same time high capture success rates. Indeed, wild *Pipistrellus kuhli* adjust their flight speed to the task so as to balance energetic costs. They fly faster when commuting (*i.e.* closer to the speed required for minimizing energy expenditure per distance traveled) and slower during hunting (*i.e.* closer to the speed required for minimizing energy expenditure per time spent flying). This indicates that bats tailor their flight speed in a context-dependent manner to optimize energy efficiency (Grodzinski et al., 2009). Sensory sampling studies from the wild show that bats can emit calls up to 220 times per second during buzzing to successfully capture aerial prey (Ratcliffe et al., 2013). Since sensory sampling must be tied to wingbeats to be efficiently produced, sampling faster might result in either less efficient flight (if wingbeats are adjusted to sensory sampling) or less efficient sound production (if sound production is decoupled from wingbeats) (Jones, 1994, 1999; Waters and Wong, 2007). Here, we investigate how bats resolve that trade-off between biosonar information flow and energetic efficiency of flight and sound production during natural foraging behaviors. We hypothesize that wild bats have distinct sensory-motor combinations of wingbeat, call rate, and call intensity adapted to the different behavioral task of either aerial capture or commuting flight. To test this, we used high-resolution biologging tags to measure biosonar source levels and sampling rates as a function of wingbeat cycle in greater mouse-eared bats as they hunt and orient in the wild during nightlong foraging trips.

### Sensory update rates are strictly coupled to wingbeat only when commuting and searching for prey

**Figure 1:**
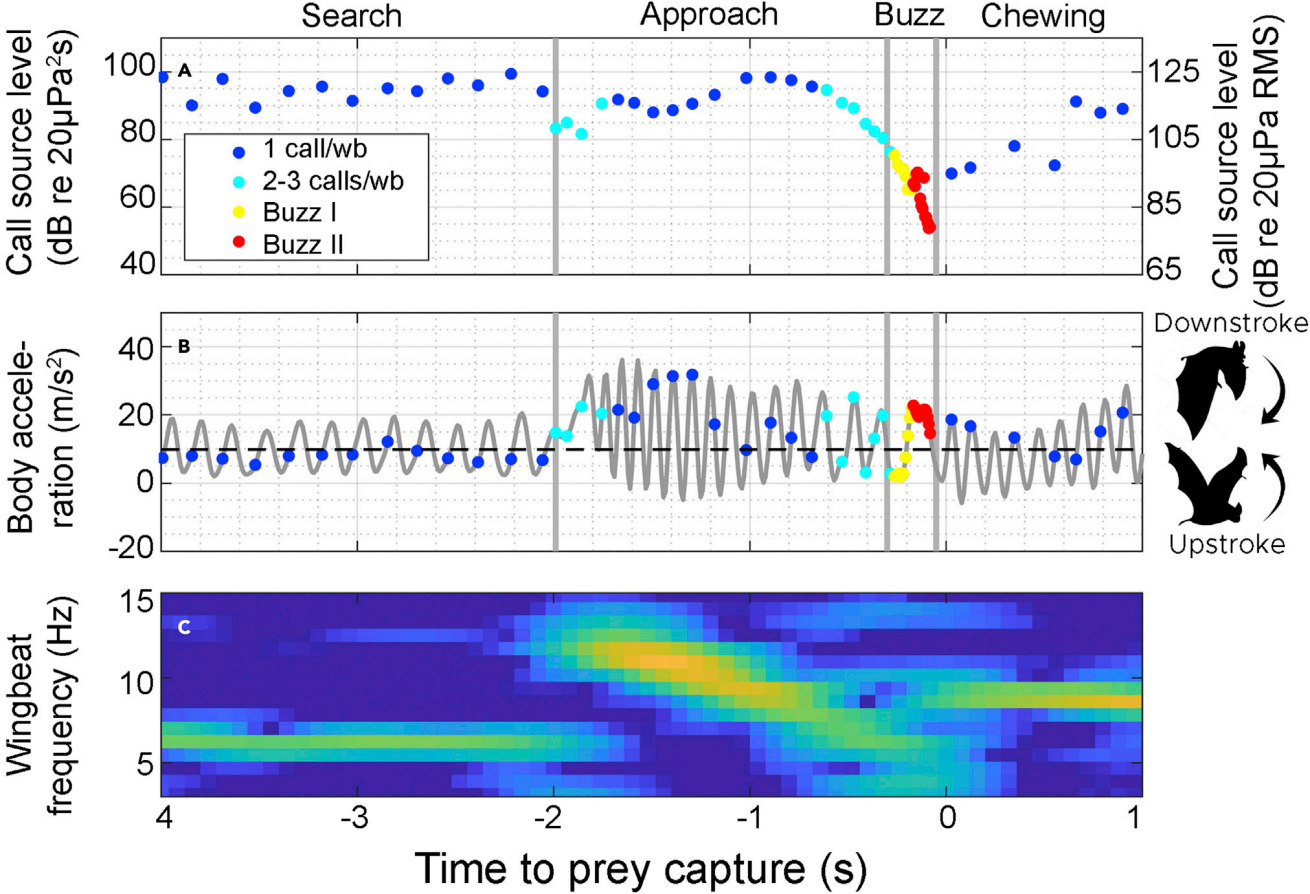
Example of the coupling and de-coupling between echolocation calls and movement during searching for, and capturing of, an aerial insect (A) Call output levels (left-hand axis: measured in energy flux density, right-hand axis: RMS sound pressure level) vary over time and strongly decrease toward the capture. Colors indicate the number of calls emitted per wingbeat (wb) cycle. (B) Wingbeats generate sinusoidal acceleration signals that are logged by the tag (gray). Call emissions are initially coupled one to one to wingbeat cycle (blue) in the search phase but increase to 2-3 calls per wingbeat (light blue) in the approach phase. In the buzz (buzz I, yellow, and buzz II, red), more than eight calls are emitted per wingbeat. (C) The instantaneous wingbeat frequency and amplitude during the course of the capture. Two seconds before the capture, the wingbeat frequency increases dramatically from 6 to 13 Hz presumably marking the adjustments in flight behavior after prey detection. After prey capture, the bat transitions to a wingbeat rate of 9 Hz while chewing. The spectrogram was produced with an Fast Fourier Transform and window length of 128, an overlap of 100 samples at an accelerometer sampling rate of 100 Hz and a dynamic range of 30 m/s^2^.

**Figure 2:**
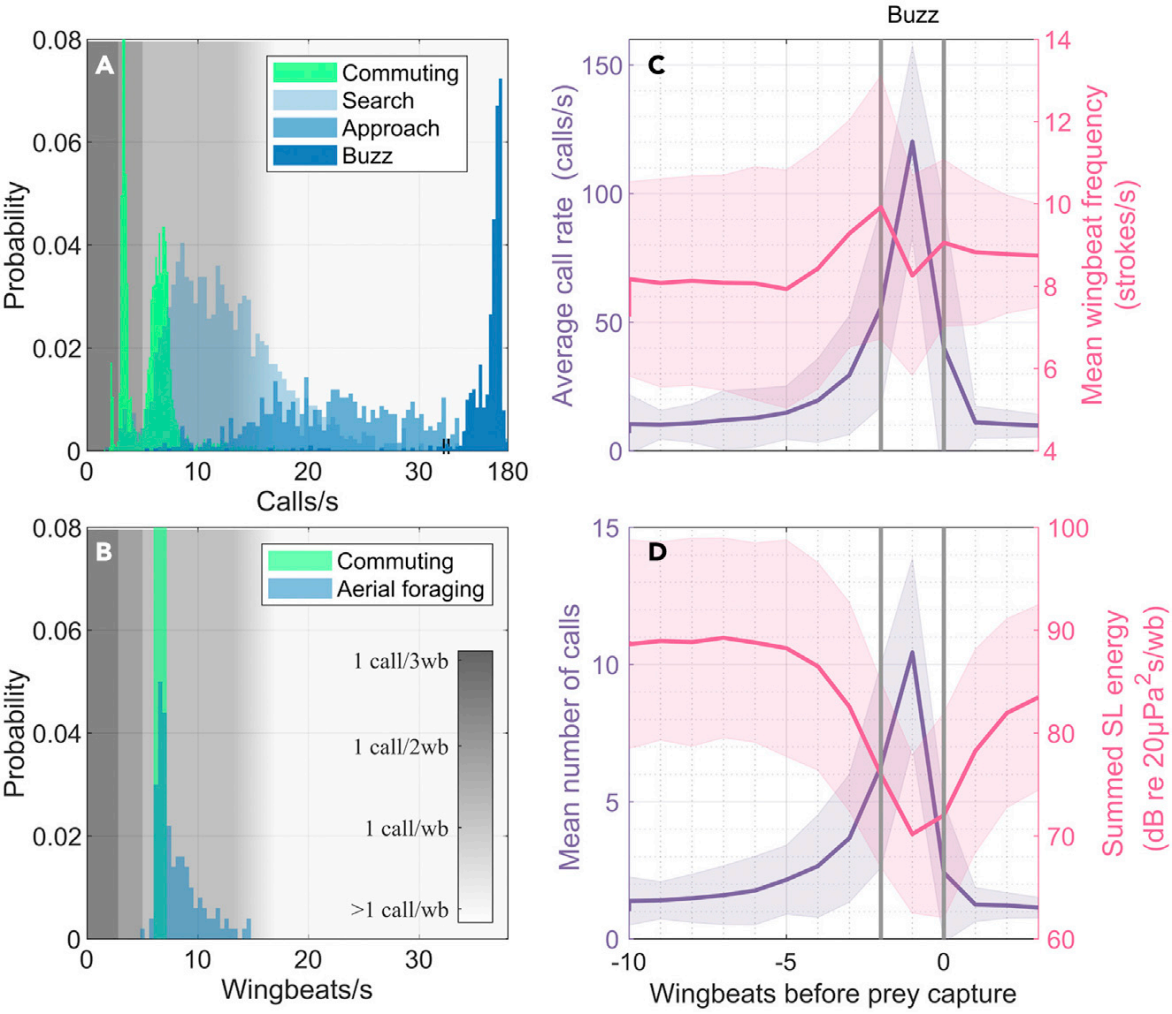
Call emissions are tied to wingbeats during commuting flight but decouple during aerial approach and capture of insects (A) Distribution of the number of calls emitted per second, with x axis broken at 32 calls per second to show the buzz calls. (B) Distribution of the number of wingbeats per second, as extracted from the peak of the z axis acceleration. (N = 10 bats, 11 commuting flights with durations of 100 s per bat and 483 aerial captures of ~3 s per capture). (C) Development of the call rate (purple) and the mean wingbeat frequency (pink) during the last ten wingbeats before capture. The call rate and the wingbeat frequency increase toward the capture. (D) Development of the mean number of calls per wingbeat (purple) and the emitted summed energy of all calls within each wingbeat (pink), expressed in terms of energy flux density (dB re 20μPa^2^s). The summed energy decreases toward the capture despite an increasing number of calls per wingbeat. The buzz phase starts at on average about one-two wingbeats before capture (gray, vertical lines). Means are depicted by solid lines while the shaded regions indicate the standard deviation. (N = 10 bats, 483 captures).

To quantify the coupling between sensory sampling and biomechanics, we equipped ten female greater mouse-eared bats (*M. myotis*) with sound and motion recording tags (Stidsholt et al., 2018) during one night of activity. The tagged bats commuted to foraging sites and captured on average 48 (35 standard deviation) insects on the wing as well as various insects on the ground during one night of foraging (Table S1). We first investigated the relationship between repetition rates and wingbeat frequencies when commuting and hunting. When commuting or searching for prey, the bats employ a stereotyped flight gait with wingbeat frequencies of around 7 Hz (∼140–160 ms wingbeat period; Figures 1 and 2B). Bats of this size using wingbeat frequencies of ∼7 strokes per second (Figures 1C and 2B) commute at an average flight speed of 7 m/s (calculated using a weight of 34 gram derived from a bat weight of 30 g and a tag weight of 4 g (Von Busse et al., 2013)). A flight speed of 7 m/s for this size bat likely minimizes the cost of transport during commute (Rayner, 1987). In this behavioral mode, the call intervals are closely tied to the wingbeats with calls emitted on either every, every other, or every third wingbeat (Figure 2A, green). Sensory sampling during commute is therefore comparatively slow and strictly coupled to the wingbeat cycle of the animal.

While call emissions are coupled to wingbeats in a one-to-one fashion when searching for prey (Figures 1B, 2A, and 2B) (Schnitzler and Kalko, 2001), this coupling breaks down during the course of prey capture. Upon prey detection, the bats transition into an approach phase with an increased call rate (Figure 2C, purple). This increase is partly mediated by an increase in the wingbeat rate (Figures 1B, 1C, and 2C, pink), reflecting the kinematic demand to orient toward the prey, but the higher call rate is mainly achieved by an increase in the number of calls emitted per wingbeat (Figures 1B and 2D, purple). When buzzing during the final stage of prey capture, the bats emit numerous weak calls with short call intervals leading to on average 11 calls emitted per wingbeat cycle (Figure 2D, purple). The average buzz duration is ∼100 ms (Figure S1), which is similar to the duration of the last wingbeat (Figure S1) meaning that bats extend their buzz call emissions throughout an entire wingbeat cycle. This observation provides the first quantitative field validation of earlier laboratory (Lancaster et al., 1995; Moss et al., 2006) and field (Kalko, 1994) studies suggesting that bats transition from full wingbeat sensorimotor coupling (search and commute) to partial (approach) or complete (buzz) uncoupling during hunting (Moss et al., 2006), thereby achieving a 20 fold increase in the rate of sensory information across these stages of echo-guided prey capture. We next investigated how these varying sampling rates affect the sensory update rates per distance traveled when bats fly at fast speeds in the wild.

### Information flow per distance traveled during capture changes dramatically in echolocating animals

To compare the relationship between sensory flow and speed across echolocating animals varying in size, speed of locomotion, and maneuverability, we calculated the sensory update rate per distance and body length traveled for bats and toothed whales. During search, the tight sensorimotor coupling in bats results in maximum one sensory update per meter flown. This is comparable to the sensory update rate per distance traveled of searching sperm whales (2 clicks/sec, 2 m/s swimming speed (Madsen et al., 2002); Table 1): a predator more than two orders of magnitudes longer and six orders of magnitude heavier. This is a surprise as small animals should use higher sensory sampling because they perceive temporal changes on finer time scales and have a higher maneuverability (Healy et al., 2013) compared to large animals traveling at the same speed. To account for these differences across animals varying dramatically in size, we compared sensory update rate of bats and toothed whales per body length traveled. When accounting for body length using a size-specific sampling rate, bats receive just one sensory update per ten body lengths traveled while searching (Table 1). This is an extremely low sensory update rate for a small animal flying at a high speed (Healy et al., 2013) (Table 1). In comparison, echolocating toothed whales use a >200 times higher size-specific sampling rate when searching than bats (Table 1). Thus, the cheap sound production system uncoupled from other biomechanical processes of toothed whales supports much higher redundancy in sensory scenes relative to their maneuverability.

**Table 1:** Sensory update rate across echolocator

Species	Body length (m)	Sensory update rate (s^−1^) Commute capture		Speeds (m/s) Commute capture		Sensory updates per distance traveled (m^−1^) Commute capture		Sensory updates per body length traveled (m^−1^) Commute capture	
Tagged bats	0.07	7	150	7	2^2^	1	75	0.07	5
Sperm whale	16	2^a^	50^b^	2^c^	4^c^	1	13	16	200
Harbor porpoise	1.5	25^d^	300^e^	1^f^	2^g^	25	150	38	225

Sensory update rates during “commute” and “capture” mode for different echolocating animals calculated per second, per distance traveled, and per body length traveled.

a Madsen, P.T., Payne, R.S., Kristiansen, N.U., Wahlberg, M., Kerr, I., and Møhl, B. (2002). Sperm whale sound production studied with ultrasound time/depth-recording tags. J. Exp. Biol. 205, 1899–1906.

b Teloni, V., Mark, J.P., Patrick, M.J.O., and Peter, M.T. (2008). Shallow food for deep divers: Dynamic foraging behavior of male sperm whales in a high latitude habitat. J. Exp. Mar. Bio. Ecol. 354, 119–131.

c Amano, M., and Yoshioka, M. (2003). Sperm whale diving behavior monitored using a suction-cup-attached TDR tag. Mar. Ecol. Prog. Ser. 258, 291–295.

d Ladegaard, M., and Madsen, P.T. (2019). Context-dependent biosonar adjustments during active target approaches in echolocating harbor porpoises. J. Exp. Biol. 222.

e DeRuiter, S.L., Bahr, A., Blanchet, M.-A., Hansen, S.F., Kristensen, J.H., Madsen, P.T., Tyack, P.L., and Wahlberg, M. (2009). Acoustic behavior of echolocating porpoises during prey capture. J. Exp. Biol. 212, 3100–3107.

f Naito, Y., Kato, A., Otani, S., Naito, Y., Kato, A., and Kawamura, A. (1974). Diving behavior and swimming speed of a free-ranging harbor porpoise, *phocoena phocoena*. Mamm. Species 16, 811–814.

g Wisniewska, D.M.M., Johnson, M., Teilmann, J., Rojano-Doñate, L., Shearer, J., Sveegaard, S., Miller, L.A.A., Siebert, U., and Madsen, P.T. (2016). Ultra-high foraging rates of harbor porpoises make them vulnerable to anthropogenic disturbance. Curr. Biol. 26, 1441–1446.

Assuming that perceptual time constants are similar between these species, it follows that bats rely on very sparse sensory inputs to detect and identify prey and to guide motor patterns and decision-making when commuting. We posit that this sparse sensory input might be the result of the (bio) physical constraints of a fast-flying echolocator in air that must couple call rates to a relatively low wingbeat rate to minimize the energy expenditure of echolocating. Despite this sparse sensory input and the very limited range of ultrasonic echolocation in air compared to ultrasound in water (Madsen and Surlykke, 2013), their powerful calls enable the bats to still sample the same volume of air multiple times with successive calls (Stidsholt et al., 2021) apparently providing sufficient sensory information to navigate and avoid obstacles. In concert with acute spatial memory in known habitats (Genzel et al., 2018; Ratcliffe et al., 2005), this low information redundancy appears to be sufficient for routine navigation. However, such slow sensory sampling for searching bats might result in lower prey detection rates and more reactive sensory motor operation in comparison to toothed whales (Hein and McKinley, 2013). If so, we speculate that bats may compensate by capturing prey with high success rates (Stidsholt et al., 2021) or by extracting more information from each sensory input than toothed whales due to the higher time bandwidth product of their echolocation calls (Woodward, 1953). Such low sampling rates coupled to wingbeats may be speculated to explain the evolution of their complex calls and high-level auditory processing that potentially increase information extraction for each call-echo pair (Corcoran and Moss, 2017).

During the final stage of capture, the tagged bats combine faster sampling with a slower flight speed resulting in a 70-fold increase in size-specific sampling rate in comparison to the search phase. This suggests that high temporal update rates may be necessary for capturing aerial prey in a three-dimensional space, as has recently been proposed in different taxa of visually hunting predators (dragonflies, flies, small birds (Boström et al., 2017)). The high size-specific update rates in toothed whales during commute and capture may reflect that whales due to their cheap sound production (Foskolos et al., 2019) might over-sample their surroundings, thereby supporting better discrimination of their prey and allowing faster and more precise guidance of their less maneuverable bodies compared to the agile aerial hunters (Madsen and Surlykke, 2013). Due to the large variations in sensory update rates per body length traveled in commuting and hunting bats, we next investigated how the emitted energy of calls and thereby detection range varied with these different sampling strategies.

### Decoupling calls from wingbeat phase allows faster calling but at weaker levels

**Figure 3:**
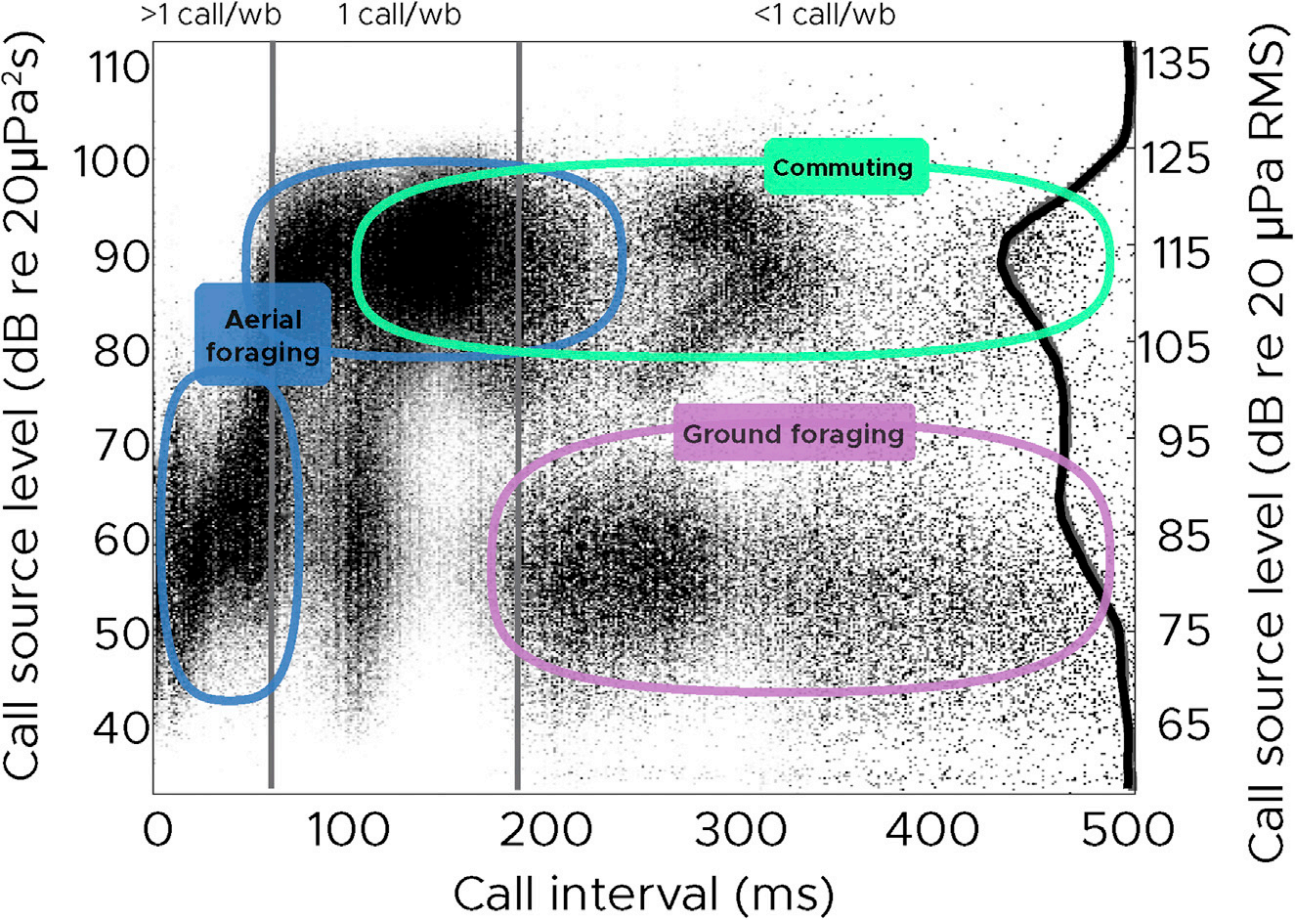
Call source levels as a function of call intervals Distinct combinations of source levels and call intervals represent different behavioral modes: Commuting flight (green) is characterized by intense call source levels (SLs) and call intervals of either ~150 ms (i.e., every wingbeat), ~300 ms (i.e., every second wingbeat), or ~450 ms (i.e., every third wingbeat). In aerial hawking mode (blue), the bats search for prey by using intense call SLs emitted every wingbeat (~150 ms call interval). As they transition into the approach phase, they reduce call interval by emitting two calls per wingbeat (~75 ms). The last part of the approach phase and the buzzes are characterized by SLs below 70 dB on an energy basis and short call intervals that are uncoupled with wingbeat. The reduction of call source level takes place within a narrow range of call intervals between 60 and 70 ms. When the bats glean insects off the ground (purple), they emit calls with low SLs but with long call intervals (~200-500 ms). The bats do not produce loud calls with short intervals of less than ~50 ms, and no weak calls at call intervals around 150 ms, i.e., emitted every wingbeat. The black line to the right marks the call source-level distribution for all bats measured in RMS. The call source level is quantified as energy flux density (left-hand axis) and as RMS (right axis) here approximated by adding 25 dB (corresponding to a fixed 3 ms call duration) to the call levels in EFD to facilitate comparison to the literature. (N = 10 bats, one full night of foraging per bat, 6 x 10^5^ calls)

The energy needed to produce sound in laryngeal-vocalizing bats is delivered by airflow (Suthers and Fattu, 1973). We therefore tested the hypothesis that bats can produce a maximum amount of sound energy per wingbeat that is constrained by the kinetic energy in the exhaled airflow. This energy can in principle be used to produce either one loud or several weaker calls. It therefore follows from this hypothesis that the summed call energy flux density (EFD, i.e., taking both the number of calls, call levels and their durations into account) per wingbeat should approach a constant. The highest call levels are emitted in commute (Figure 3, green) and search flight (Figure 3, blue), where bats emit a maximum of one call per wingbeat with call intervals of 100 ms or more. Summed call energy levels do not decrease significantly when up to two calls per wingbeat are emitted which supports previous laboratory findings (Waters and Wong, 2007). However, the summed call levels decreases when bats emit more than two calls per wingbeat (call intervals below 70 ms, Figure 3, blue). This causes an up to 100x decrease (−20 dB) in the summed call energy of all calls per wingbeat (Figure 2D, pink) during the approach and buzz phases, despite the increasing number of calls emitted per wingbeat (Figure 2D, purple). The bats therefore appear to under-utilize the available energy when calling at high rates. The disproportionately low source levels of greater mouse-eared bats when emitting more than two calls per wingbeat may have several explanations. Bats may actively call faintly to reduce sensory volumes, thus simplifying their sensory scenes during prey tracking (Stidsholt et al., 2021), or to maintain echo levels in a dynamic range suited for their hearing system at close ranges where they call at higher rates (Denzinger and Schnitzler, 1998). Alternatively, the spreading of the calls over a larger proportion of the wingbeat cycle in the buzz may make sound production less efficient causing the 100x (−20 dB) reduction in summed energy output per wingbeat cycle (Figure 2D, pink). Irrespective of whether the reduction in call levels is driven by physical constraints or a need to simplify the acoustic scene to facilitate sensory processing, faster sampling during hunting unequivocally involves weaker echolocation calls spread over more of the wingbeat cycle.

**Figure 4:**
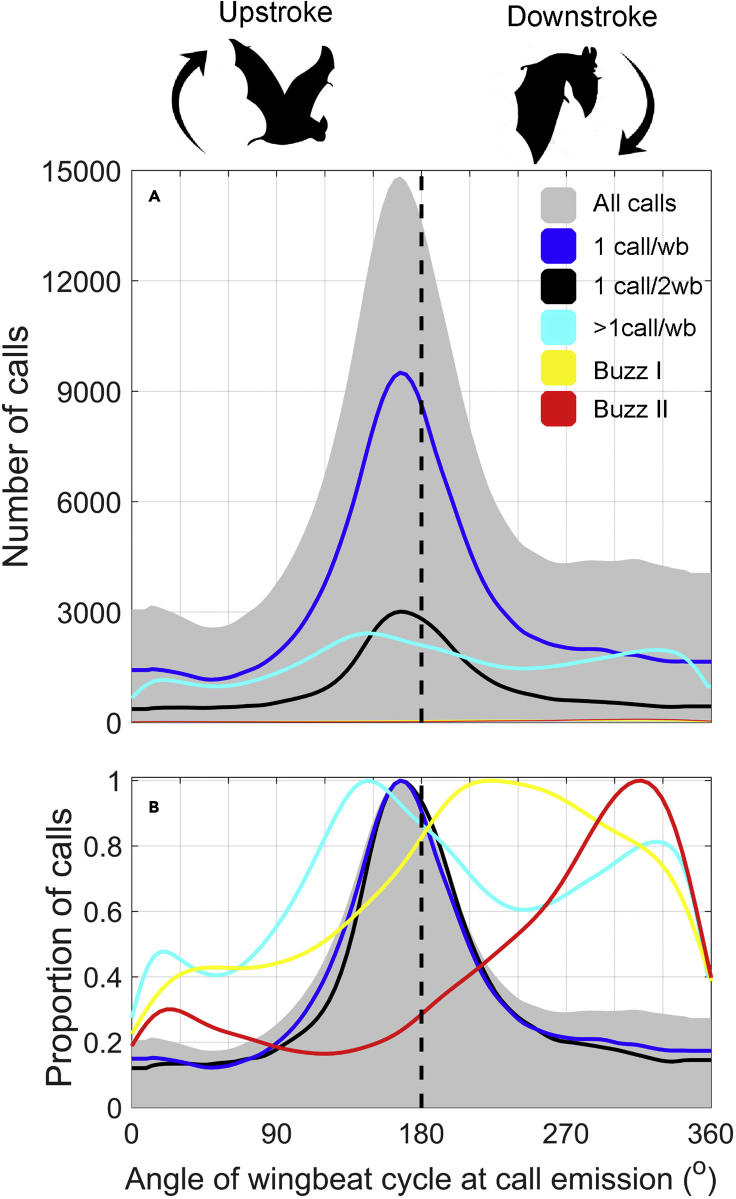
Timing of intense calls is synchronized to wingbeat phase The timing of the calls to the wingbeat phase is plotted in actual numbers (A) to illustrate the true proportions of calls and in normalized values (B) to visualize the coupling to the wingbeat phase. Calls are strictly coupled to wingbeat phase when commuting and more loosely coupled when foraging. Calls are divided into groups based on their call intervals: All calls from all tagged individuals (light gray patch). Calls with call intervals between 200 and 400 ms (1 call per 2 wingbeats, black), between 100 and 200 ms (1 call per wingbeat, dark blue), between 14 and 100 ms (above 1 call per wingbeat, light blue), between 8 and 14 ms (buzz I, yellow), and between 5 and 7 ms (buzz II, red). Upstroke is defined from 0 to 180°. (N = 10 bats, one full night of foraging per bat, 6 x 10^5^ calls). Some of the spread in apparent production phase may be due to estimation noise in inferring wingbeat angle from the measured acceleration signals.

To uncover how bats balance energy expenditure and sensory update rates during commuting and foraging, we next investigated where in the wingbeat cycle call emissions occur. If sound production efficiency is greatest at the end of the upstroke, call emissions would be expected to occur in the range of 160–180° with respect to a wingbeat cycle beginning at the start of the upstroke (0°). In keeping with that prediction, we find that the tagged bats emit most calls with a phase symmetrically centered around 176° of the wingbeat cycle (quartile range: 135-235°, Figures 4A and 4B, gray patch), corresponding to the last part of the upstroke to the beginning of the downstroke where the wings are pointing upwards. During commute and search flights, calls are likewise emitted at the last part of the upstroke (Figure S2) and the first part of the downstroke and occur either every or every second wingbeat (Kalko and Schnitzler, 1989; Schnitzler et al., 1987) (Figures 4A and 4B, dark blue and black). When commuting or searching, bats only produce high call source levels (Figure 3) with mean source levels of 107 (quartile range: 93 to 115) dB re 20 μPa RMS. Most of their search calls are therefore emitted with source levels close to or below the 110 dB re 20 μPa RMS limit associated with an increase in metabolic rate for Nauthusis's pipistrelle bats (*Pipistrellus nathusii*) (Currie et al., 2020). This indicates a strong sensory demand for intense calls that provide large sensory volumes but by synchronizing these search and commute calls with upstrokes bats can likely produce them in-expensively but at inherently low rates as dictated by their stable flight gaits.

When transitioning into the approach phase with multiple calls per wingbeat, call emissions occupy a larger fraction of the wingbeat cycle and therefore extend beyond the upstroke also into the end of the downstroke (Figure 4, light blue, Figure S3) (Kalko and Schnitzler, 1989; Koblitz et al., 2010). These calls are timed to the wingbeat cycle in a similar fashion as the dyads and triplets emitted during a landing exercise in *Eptesicus fuscus* (Koblitz et al., 2010) highlighting a stereotyped pattern across different species of bats. The timing of buzz I and II calls is shifted even further from the peak, being mainly timed to the first and middle part of the downstroke (Figure 4B, yellow and red). Buzz calls thus are regularly emitted after the upstroke (Figure 4B, light blue). In the buzz, bats must therefore extend the exhalation throughout the downstroke to continuously produce calls with repetition rates above 160 calls/second. Bats therefore actively choose to override the wingbeat cycle when fast streaming of echo information is needed by decoupling sound emissions and breathings from the upstroke. Interestingly, the decoupling is still not complete (see clear peak of buzz calls at ∼320°), which likely reflects a stereotyped motor pattern just before prey capture.

### Costs of sound production may have put an evolutionary premium on low biosonar sampling rates in bats

The need for fast sensory sampling during the approach and buzz phases results in calls being emitted well outside of the optimal wingbeat phase implying reduced sound production efficiency for these calls. However, the energy per buzz call is on average 1000 times lower (−30 dB) than search call energy (Figure 3, blue boxes), and buzz calls comprise <2% (Figure 4A, red and yellow) of the total emitted calls throughout a night of foraging, suggesting small absolute costs of such decoupling. However, if we use a less conservative estimate by including all approach calls emitted in a sub-optimal wingbeat phase (<90 and >270°), these call emissions comprise 13% of all calls and have a median call level 12 dB louder than the buzz calls indicating that wingbeat-decoupled sound emissions during hunting may incur added energetic costs to the bats. While such energetic implications perhaps are specific to the species and study conditions, we consider it parsimonious that they are representative for other frequency-modulated, insectivorous bats. Future studies should address how the relationship between call rate, levels, and wingbeat phase varies in bats feeding exclusively on aerial prey or in bats using longer duration calls such as constant frequency (CF) calls. These CF bats would be expected to break the coupling between wingbeat and call emissions more frequently possibly incurring larger energetic costs.

In contrast to the low relative number of buzz calls in our tagged bats, up to 75% of all emitted clicks in echolocating beaked whales are buzz clicks (Madsen et al., 2013). However, buzz clicks in toothed whales are produced by a pneumatic sound production system that likely has a very low energetic cost (Foskolos et al., 2019) providing cheap sensory sampling despite extremely high sampling rates. Thus, for bats, the frugal use of short epochs of fast but weak sonar sampling decoupled from wingbeats allows bats to achieve high information update rates to optimize auditory streaming of their complex environments during critical hunting moments at little additional cost to their overall costs of sound production. Conversely, it may be speculated that the bats' low sampling rates during commute and prey search have been selected for because of the high energetic cost if these calls were uncoupled from the wingbeat (Speakman and Racey, 1991). The much lower biosonar sampling rates per traveled body length in bats compared to toothed whales have previously been ascribed to the slower sound speeds in air (Madsen and Surlykke, 2013). Our data, in contrast, support the interpretation that wingbeats restricting cheap and powerful vocalizations to specific phases of the wingbeat cycle have been a major driver underlying slow sensory sampling rates of bats (Jones, 1999). We speculate that these biomechanical constraints have put an evolutionary premium on complex echolocation signals (Woodward, 1953) and movement patterns (Hedenström and Christoffer Johansson, 2015) in bats to make the most of the infrequent sampling to maximize echo information while avoiding obstacles. The converse is true for toothed whales that have very cheap click production (Noren et al., 2017) decoupled from both locomotion and breathing (Foskolos et al., 2019), allowing them to sample orders of magnitude faster per body length traveled with simple biosonar signals.

### Limitations of the study

This study did not include measurements of energy consumption of the wild bats due to methodological limitations. This would be crucial in future studies to address the direct energetic requirements and costs of sensory acquisition in wild bats.

## STAR★Methods

### Key resources table

**Table:** 

REAGENT or RESOURCE	SOURCE	IDENTIFIER
**Deposited data**		

Analyzed data	This paper; Mendeley Data	https://doi.org/10.17632/9zfncc3t8j.1
Code	This paper; Mendeley Data	https://doi.org/10.17632/9zfncc3t8j.1

**Experimental models: Organisms/strains**		

Female Greater-mouse eared bats	Bulgarian authorities (MOEW-Sofia and RIOSV-Ruse)	N/A

### Resource availability

#### Lead contact

Further information and requests for resources should be directed to and will be fulfilled by the lead contact, Laura Stidsholt (laura.stidsholt@bio.au.dk).

#### Materials availability

This study did not generate new unique reagents.

#### Data and code availability

• Data generated in this study have been deposited at Mendeley Data (https://doi.org/10.17632/9zfncc3t8j.1) and is publicly available.
• The original code has been deposited at Mendeley Data (https://doi.org/10.17632/9zfncc3t8j.1) and is publicly available.
• Any additional information required to reanalyze the data reported in this paper is available from the lead contact upon request.

### Experimental model and subject details

During the field seasons of 2017, 2018 and 2019, we tagged and recaptured 10 female, post-lactating greater mouse-eared bats (*Myotis myotis*, Vespertilionidae) at the Orlova Chuka cave, Ruse, Bulgaria, under permit from the relevant authorities (MOEW-Sofia and RIOSV-Ruse, permit numbers: 721/12.06.2017, 180/07.08.2018 and 795/17.05.2019).

### Method details

#### Experimental setup

Bats were caught with a harp trap at Orlova Chuka cave, close to Ruse, NE-Bulgaria, in the early mornings as they returned to the roost. The bats were kept at the Siemers Bat Research Station in Tabachka to measure the forearm lengths, CM3 and body weights (Table S1). Bats weighing above 28 grams were tagged and released the following night between 10-11 p.m. at a field 8 km from the roost (Decimal degrees: 43.6220, 25.8649. The tags were wrapped in balloon rubber for protection and glued to the fur on the back between the shoulders with skin-bond latex glue (Ostobond, Montreal, Canada). The microphone on the tag was located at the center of the body approximately 10 cm behind the head of the bat. The bats spent 2 to 14 days equipped with the tags until they were recaptured at the cave or until the tags detached from the bats and fell to the ground below the colony. Upon recapture, the bats were weighed and checked for any sign of discomfort from the tagging before they were released back to the colony.

#### On-board tag and its effect on the bats

The acoustic tag used for these studies recorded audio with an ultrasonic microphone (FG-23329, Knowles Electronics, Itasca, IL, USA) and sampled the bat’s behavior by synchronized tri-axial accelerometers and magnetometers (Stidsholt et al., 2018). The audio was recorded at a sample rate of 187.5 kHz (16 bit resolution) and with a clip level of 121 dB re 20 μPa. The microphone output was filtered with a one-pole, 10 kHz high-pass filter and an antialiasing filter of 80 kHz before sampling. The accelerometers sampled at 1000 Hz (16 bit resolution, 8 g clip level) with a 250 Hz anti-alias filter, while the magnetometers sampled at 50 Hz. The tags including radio transmitters weighed between 3.5 - 3.9 grams in the field (Table S1) and therefore weighed 11 to 14 % of the body mass of the bats. The bats on average lost ∼2.5 g during the tagging period which is less than the average diurnal loss in body mass of 5.5 g during the one day spent at the station prior to release (Table S1). In addition, these bats caught prey up to several hundred times per night with high success rates (Table S2) suggesting that the tags did not have large effects on their ability to maneuver and catch prey as seen in previous studies (Egert-Berg et al., 2018; Stidsholt et al., 2021).

### Quantification and statistical analysis

#### Definitions of behaviors

Commuting flights (used in Figures 2A and 2B) were identified as lasting for approx. 100 continuous seconds per tag recording, where the bats were flying without attempting to catch either aerial or ground prey and with wingbeat frequencies of approximately 7 Hz. Aerial foraging attempts (used in Figures 1 and 2) were manually identified if bats emitted a buzz while they were in flight to exclude landing buzzes. In an average of ∼80% of the aerial captures, chewing sounds were audible in the recordings. The aerial captures were divided into search, approach and buzz phases. Five participants manually marked the beginning of the approach phase based on call intervals and source levels plotted against time to prey capture. Whenever three of the five participants marked the transition into the approach phase in the same time interval (+/- 120 ms), the mean value was used as the onset of the approach phase. The time of capture was defined as the emission time of the last buzz call. Buzz I was defined as calls with call intervals from 7 to 14 ms prior to buzz II; buzz II included calls with call intervals from 4 to 6 msec (Melcón et al., 2009).

#### Data analysis

Tag data were adjusted for the frequency response of the microphone and high-pass filtered by a 4-pole 10 kHz high pass Butterworth filter to extract only the echolocation calls. Accelerometer data were low-pass filtered with a delay-free linear phase finite impulse response (FIR) filter with a cut-off frequency of 30 Hz. All analyses were conducted using custom-written scripts (Matlab, 2019a, The Mathworks, Natick, MA, USA). All calls in the recordings were automatically extracted and visually inspected for correct detections. Calls were not extracted in time periods where loud sounds from *e.g.* wind or conspecific calls appeared in the recordings to avoid false detections. As the calls of the bats were emitted in a directional beam in front of the bat, the tag-recorded call levels were lower than the actual on-axis call levels. The difference between the off and on-axis call levels for this species was estimated at 14 dB (Stidsholt et al., in review). Source levels were therefore estimated by adding 14 dB to the call levels measured in energy flux density (dB re 20μPa^2^s) over a -6 dB energy window from the tag-recordings. This conversion does not take head movements into account, which may shade some calls, but not in an extend affecting our conclusions of the study.

The call source level was also quantified in RMS approximated by adding 25 dB (corresponding to a fixed 3 ms call duration) to the call levels in EFD to facilitate comparison to the literature.

#### Relationship between flight speed and wingbeat frequency

Bullen and McKenzie (2002) measured wingbeat frequencies across different flight speeds for 23 bat species and found the relationship:

Wingbeat frequency = 5.54 - 3.068∗log10(body mass) - 2.857∗log10(flight speed) [S4]

Using a body mass of 34 gram (tag-weight included) and a flight speed of 7 m/s as a mean from GPS positions when bats return to their roosts (unpublished data), the wingbeat frequency in commuting flight would be 7.3 Hz. This is close to the wingbeat frequency we found for commuting bats of 6-7 Hz.

#### Relationship between body-acceleration and angles of the wingbeat cycle

The on-board tag samples tri-axial acceleration from the back of the bat at approximately the center of gravity with the accelerometer axes following the left-hand rule (forward-right-up). To analyze the wingbeat cycle, we converted the sinusoidal acceleration in the z-dimension (heave) into phase angle from 0 to 360 degrees. The degrees 0 to 180 correspond to acceleration values below 9.82 m/s^2^, where the body with the tag is accelerating towards the ground powered by the upbeat of the wings. The degrees from 180 to 360 degree correspond to acceleration values above 9.82 m/s^2^, where the body is accelerating upwards powered by the downstroke.
