# Supplementary material for: Wild bats briefly decouple sound production from wingbeats to increase sensory flow during prey captures
Source: iScience. 2021 Jul 22;24(8):102896. doi: 10.1016/j.isci.2021.102896 (PMC8355945; doi:10.1016/j.isci.2021.102896)
Supplement: Document S1. Figures S1–S3 and Tables S1–S2 [file mmc1.pdf]

iScience, Volume *24*

Supplemental information

Wild bats briefly decouple sound production from wingbeats to increase sensory flow during prey captures

Laura Stidsholt, Mark Johnson, Holger R. Goerlitz, and Peter T. Madsen

Table S1. Summary of the weights and sizes of the tagged bats, Related to Figure 1-4. The bats were weighed (weight at capture) and measured (CM3 and forearm length) when captured in the morning and when released at night (weight at release). The difference between release and capture weights correspond to the daily weight loss while kept at the field station (Weight loss at station) due largely to digestion and water loss. Upon recapture, the bats were weighed (bat weight at recapture). If recapture weights of the bats is missing (indicated by "-"), the tags were found on the ground below the colony. The weight of the tags differed depending on the size of the battery (tag weight). The tag to body weight (%) indicates how much of the body mass of the bat the tags comprise. f: female, PL: post-lactating, L: lactating.

Bat ID	Year	Sex & status	CM3 (mm)	Forearm length (mm)	Weight at capture (g)	Weight at release (g)	Weight loss at station (g)	Bat weight recapture (g)	Tagging weight loss (g)	Tag weight (g)	Tag to body weight (%)
L17	17	f, PL	-	-	-	30	-	27.3	2.7	3.5	11.7
L1	18	f, L	-	-	37.9	31.2	6.7	29.5	1.7	4	12.8
L3	18	f, L	-	-	36	30.1	5.9	27.3	2.8	3.7	12.3
L5	18	f, L	10	66.7	35.8	30.5	5.3	28	2.5	4	13.1
L6	18	f, L	9.8	64.1	36.2	29.8	6.4	27.1	2.7	3.8	12.8
L7	18	f, L	10	64	34.8	29.4	5.4	25.1	4.3	3.6	12.2
L10	18	f, L	10	66.1	36.1	30.1	6	27.1	3	3.5	11.6
L11	18	f, PL	10.1	66.1	33.1	29.3	3.8	-	-	4	13.7
L23	19	f, PL	10	63.4	33.2	28	5.2	27.1	0.9	3.5	12.5
L24	19	f, PL	-	-	33.6	28.9	4.7	-	-	3.8	13.1

Table S2. Summary of the foraging attempts and captures of ten *Myotis myotis*. Related to Figure 2. The bats attempted to catch both aerial prey (total aerial attacks) and ground-based prey (total ground attacks).

Bat ID	Total aerial attacks	Total ground attacks
L1	12	59
L3	21	68
L5	77	11
L6	103	0
L7	71	5
L10	82	3
L11	11	179
L17	4	33
L23	23	166
L24	79	56
**Mean**	**48**	**58**
**Sum**	**483**	**580**

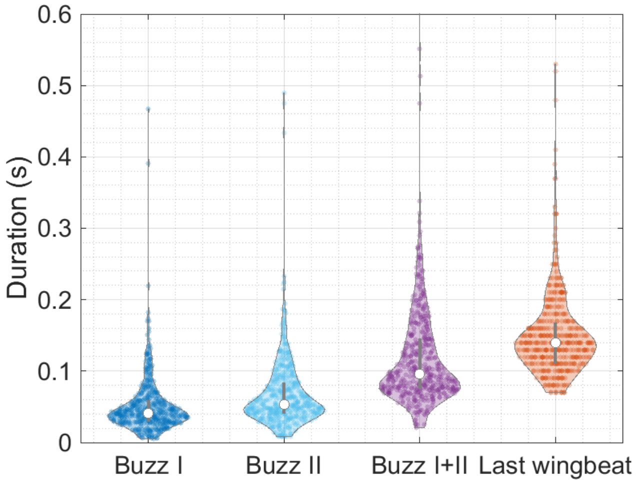

Figure S1. Duration of the buzzes and the last wingbeats. Related to Figure 1-2 & 4. The duration of the buzz and of buzz I and II components was determined for each aerial capture. Median buzz duration (buzz I+II) is 120 ms. Buzz II comprise ~70 % of the overall buzz durations. (N = 11 bats, 483 captures). The duration of the last wingbeat before the end of the buzz is plotted to enable comparison to the length of the buzz.

-

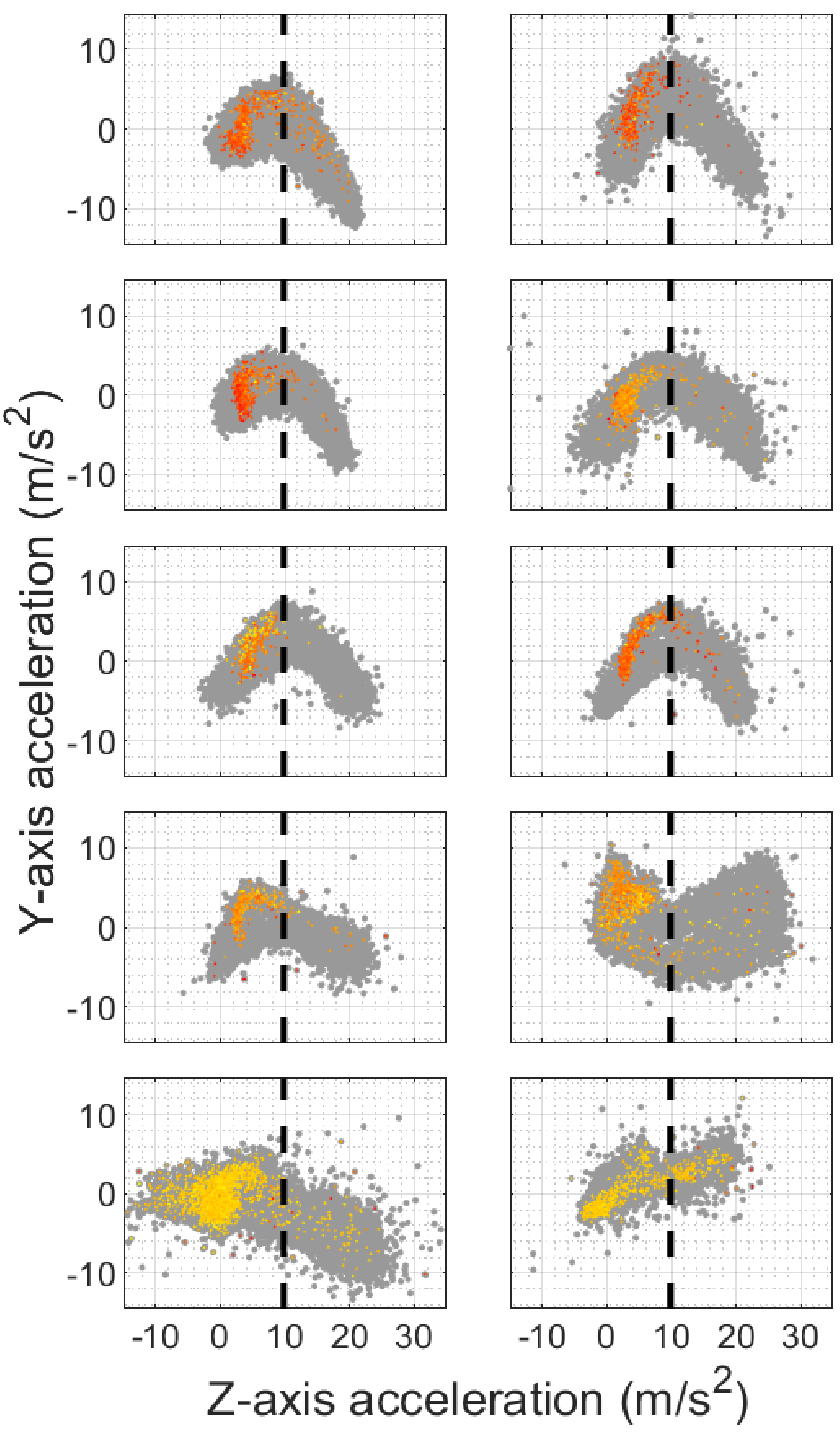

Figure S2. Lissajous plots to analyse where in the wingbeat cycle calls occur in commuting flights for each individual bat. Related to Figure 4. Each panel corresponds to 100 seconds of commuting flights per tagged bat. The Lissajous plots show the cyclic accelerometer samples during wingbeats (grey). Call

emissions (coloured dots) are plotted on top to show where in the wingbeat cycle they occur. Calls are colour-coded to the energy normalised for each plot from yellow to blue (strongest-to-weakest calls). Most calls appear at less than 9.82 m/s^2 of z-axis acceleration, i.e., during the upstroke.

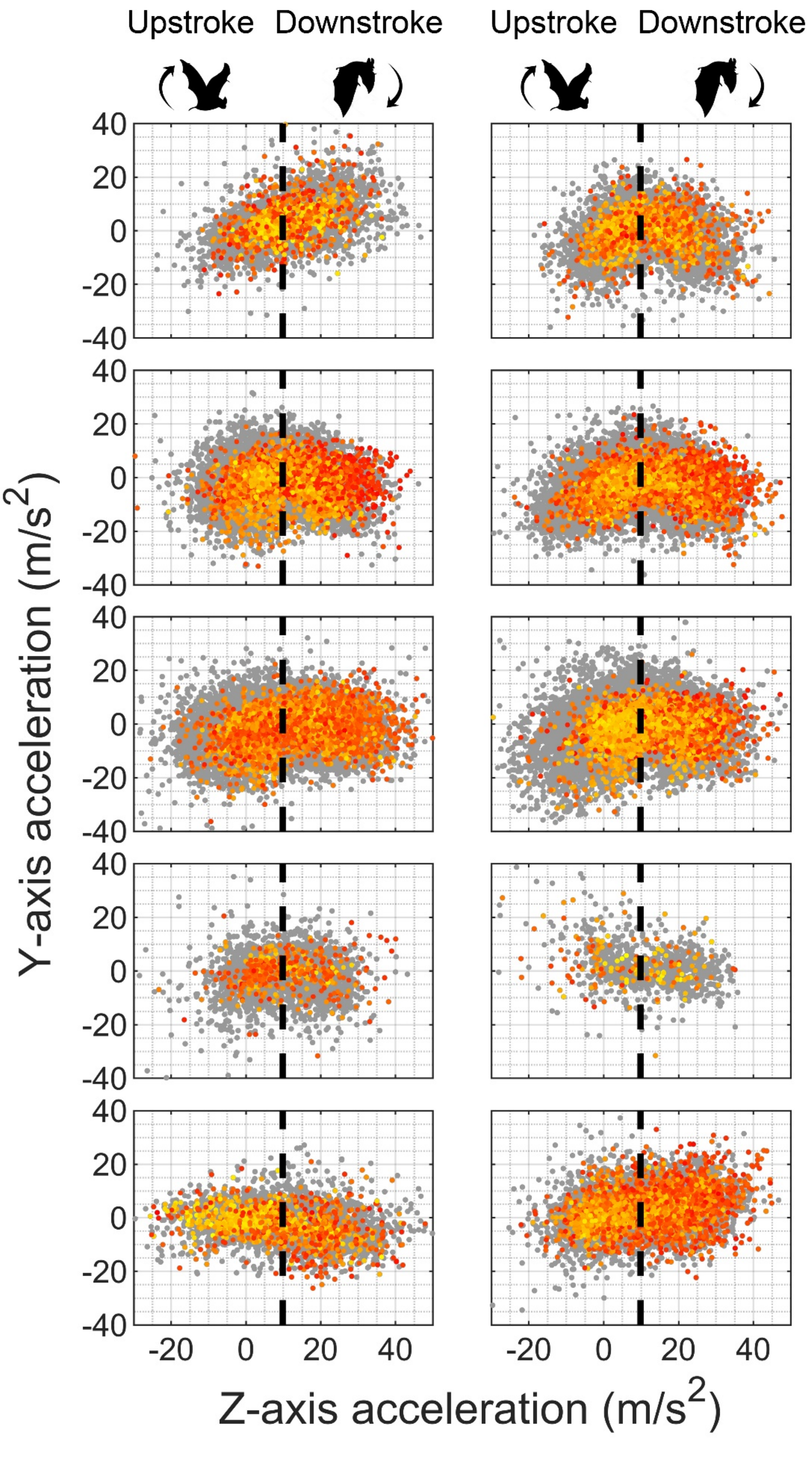

Upstroke
Downstroke
Upstroke
Downstroke
Y-axis acceleration (m/s²)
Z-axis acceleration (m/s²)
40
20
0
-20
-40
-20
0
20
40

Figure S3. Lissajous plots to analyse where in the wingbeat cycle calls occur during captures. Related to Figure 4. Each panel corresponds to all captures per tagged bat. The shape of the Lissajous plots are more circular (grey) implying a more variable gait, and the call emissions (coloured dots) are spread over the plots. Calls are therefore occurring over the entire wingbeat phase when capturing prey. Calls are colour-coded according to the normalised energy for each plot from yellow to blue (loudest-weakest calls). The calls are placed over the entire wingbeat cycle when the bats are capturing prey.
